# The Fatal Course of Pulmonary Mucormycosis: A Case Report

**DOI:** 10.7759/cureus.66018

**Published:** 2024-08-02

**Authors:** Arman Sindhu, Ulhas Jadhav, Babaji Ghewade, Jay Bhanushali, Souvik Sarkar, Pallavi Yadav

**Affiliations:** 1 Department of Respiratory Medicine, Jawaharlal Nehru Medical College, Wardha, IND; 2 Department of Obstetrics and Gynecology, Jawaharlal Nehru Medical College, Wardha, IND

**Keywords:** cystic consolidation, amphotericin-b, mucorales, fungal hyphae, bird nest appearance

## Abstract

Pulmonary mucormycosis is a rare and lethal fungal infection elicited by fungi of the order Mucorales. The disease predominantly affects immunocompromised hosts, like those with diabetes mellitus, hematologic malignancies, or those undergoing immunosuppressive therapy. We, at this moment, report a case of pulmonary mucormycosis in a 55-year-old gentleman, exemplifying the ferocity of clinical disease, diagnostic dilemmas, and rapidity of progression. A diagnosis of pulmonary mucormycosis was based on diagnostic imaging and flexible bronchoscopy. Despite aggressive antifungal and supportive treatment, the patient's condition deteriorated further, and unfortunately succumbed to cardiorespiratory arrest. This case reinforces the importance of early recognition of pulmonary mucormycosis and aggressive medical management, especially in immunocompromised patients, in salvaging lives with good outcomes and preventing the fulminant progression of the disease process.

## Introduction

Pulmonary mucormycosis is known to present a significant clinical challenge because it tends to progress aggressively and affects immunocompromised individuals. Systemic diseases leading to immunosuppression involve diabetes mellitus, recipients of organs, and malignancy under treatment [1]. The causative agents are Mucorales saprophytic fungi, which invade the lung tissue significantly when the host's defenses have been compromised. These fungi multiply aggressively within the invaded lung tissues and go ahead, causing much damage [2]. The pulmonary pathogenesis of mucormycosis occurs as inhaled fungal spores germinate in the lungs to produce hyphae that establish vascular invasion, which can cause infarction and necrosis of the pulmonary tissue. This vascular invasion lays the foundation for a host of clinical complications, among them massive pulmonary hemorrhage and its resultant failure [3]. Rapid exacerbation enforces the fact that early detection and therapeutic intervention should commence as soon as possible. However, the presentation of pulmonary mucormycosis is often nonspecific: initial symptoms include fever, cough, and dyspnea, thereby further complicating the process of timely diagnosis. Furthermore, this contributes to the diagnostic dilemma as it might overlap clinical and radiological features with other pulmonary infections such as aspergillosis [4].

## Case presentation

A 55-year-old man presented to the emergency department with complaints of a cough, breathlessness, and right-sided chest pain persisting for eight days. His breathlessness corresponded to Modified Medical Research Council (mMRC) grade III, alleviated only with rest, while his cough was productive with whitish sputum, and the chest pain was localized to the right side. He had a history of hypertension and type II diabetes mellitus for 10 years, managed with oral medications. Additionally, he had a chronic smoking history (20 pack years) and had been an alcoholic for over 25 years. Four days prior, he was admitted to a private hospital and subsequently referred to a higher center for further management. There was no other significant past medical history.

Upon examination, his pulse was 102 beats per minute, respiratory rate 34 breaths per minute, blood pressure 140/90 mmHg, and room air oxygen saturation was 88%. Auscultation revealed reduced breath sounds on the right side, with normal S1S2 heart sounds and no murmurs. Abdominal examination revealed a soft and non-tender abdomen, and central nervous system (CNS) examination showed no focal neurological deficits. He was then transferred to the intensive care unit (ICU) for further management.

**Figure 1:**
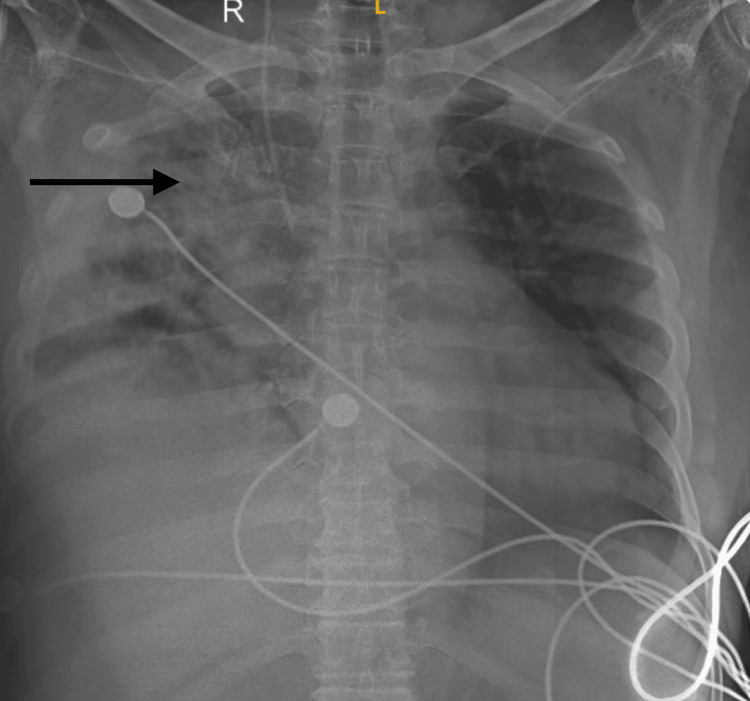
Chest X-ray showing opacities in the right upper lobe with surrounding consolidation.

Laboratory results were within normal limits, and he was initiated on antibiotics, nebulization, injectable insulin, and non-invasive ventilatory support. A chest X-ray showed heterogeneous opacity in the right lung (Figure 1).

**Figure 2:**
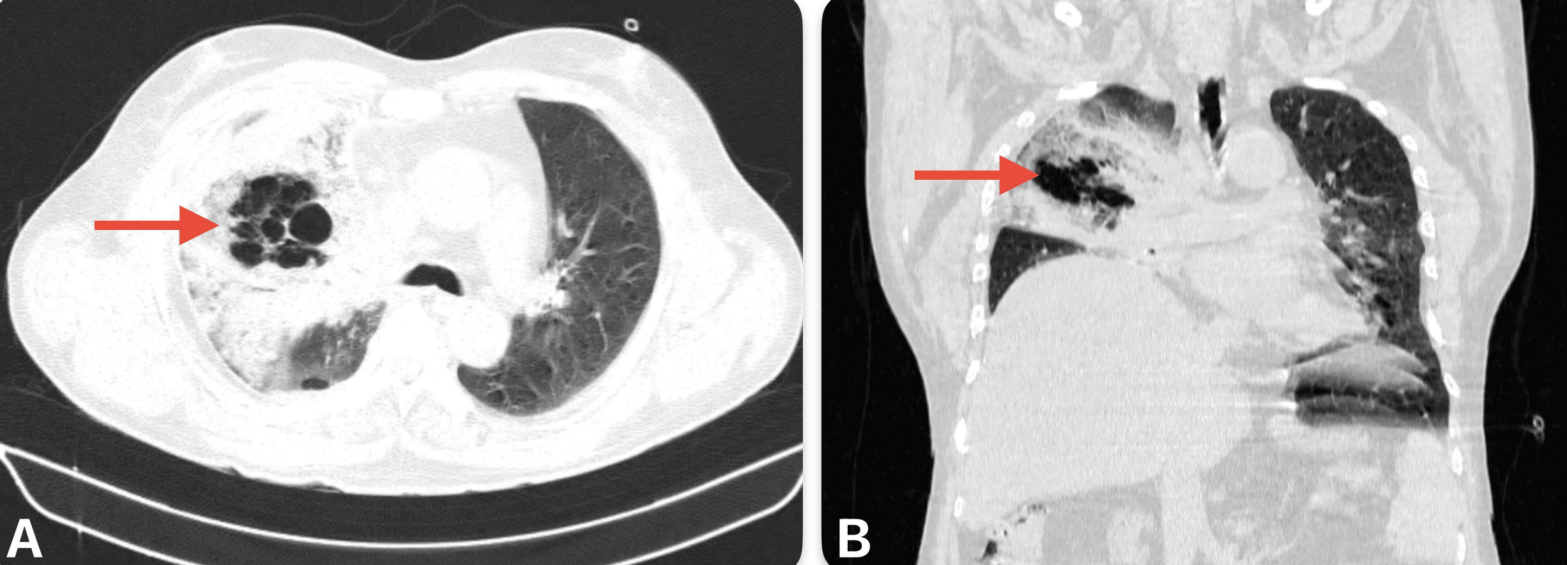
CT imaging of thorax lung window. (A) Axial view showing bubbly cystic lucencies with surrounding ground-glass opacity. (B) Coronal view showing surrounding consolidation.

**Figure 3:**
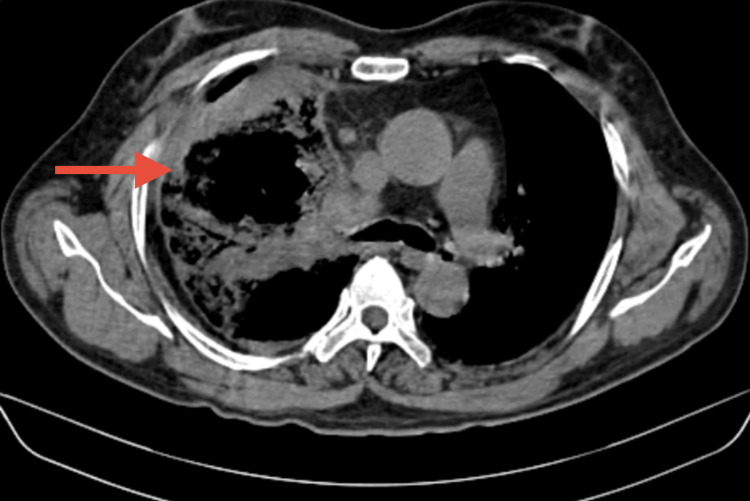
CT imaging of the thorax mediastinal place shows a cystic cavity with surrounding consolidation in the right upper and middle lobe.

A high-resolution CT scan of the thorax revealed bubbly cystic lucencies in the right upper lobe, along with surrounding consolidation and ground-glass opacities in the right upper, middle, and lower lobes. These findings were indicative of fungal pneumonia, most likely mucormycosis (Figure 2 and Figure 3).

Following the initiation of antifungal treatment, the patient developed hypokalemia, necessitating multiple potassium corrections. He experienced episodes of tachycardia, with an electrocardiogram (ECG) suggestive of multifocal atrial tachycardia (MAT), and a 2D echocardiography revealed an ejection fraction of 40%-50%, with other findings within normal limits. He was subsequently started on calcium channel blockers and beta blockers.

**Figure 4:**
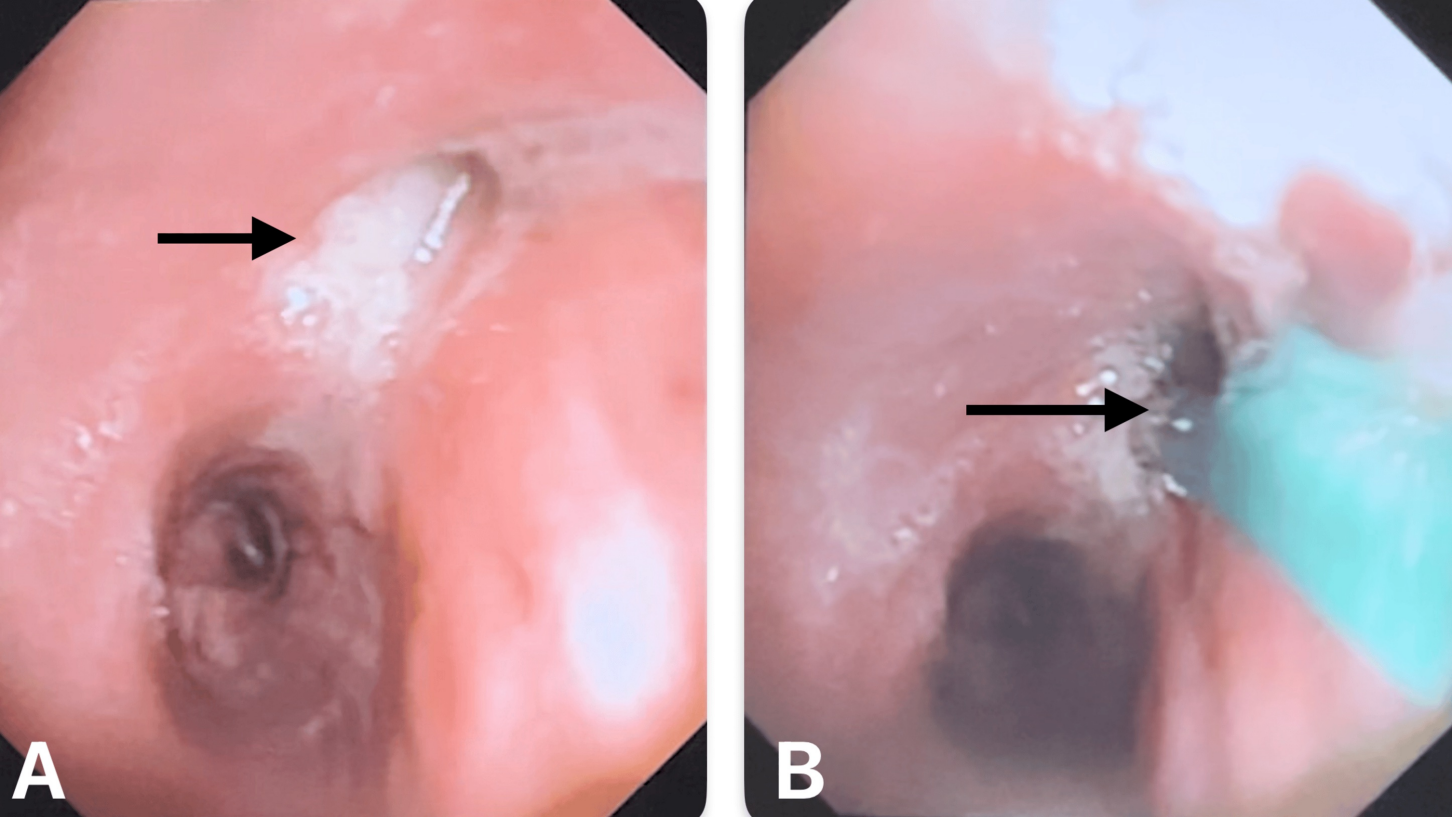
Flexible optic bronchoscopy shows (A) mucus plug impaction in the right upper lobe bronchus and (B) biopsy taken from the right upper lobe.

Flexible bronchoscopy revealed a thick mucus plug in the right upper lobe bronchus with a black necrotic lumen (Figure 4).

**Figure 5:**
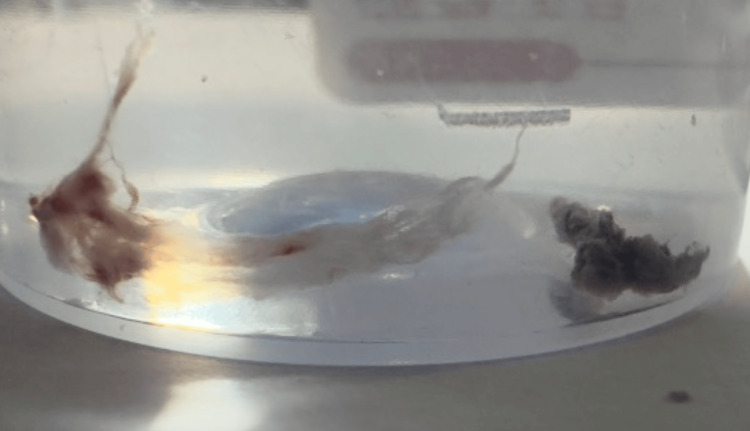
Biopsy specimen shows mucus plug and black necrotizing mucosa from the right upper lobe.

Mucus plugs and biopsies from the lumen and transbronchial lung biopsy were taken for cytology and histopathological examination (Figure 5). Sputum cultures showed growth of *Pseudomonas aeruginosa* and *Enterobacter* species, leading to a change in antibiotics according to sensitivity results. Blood cultures showed no growth. 

**Figure 6:**
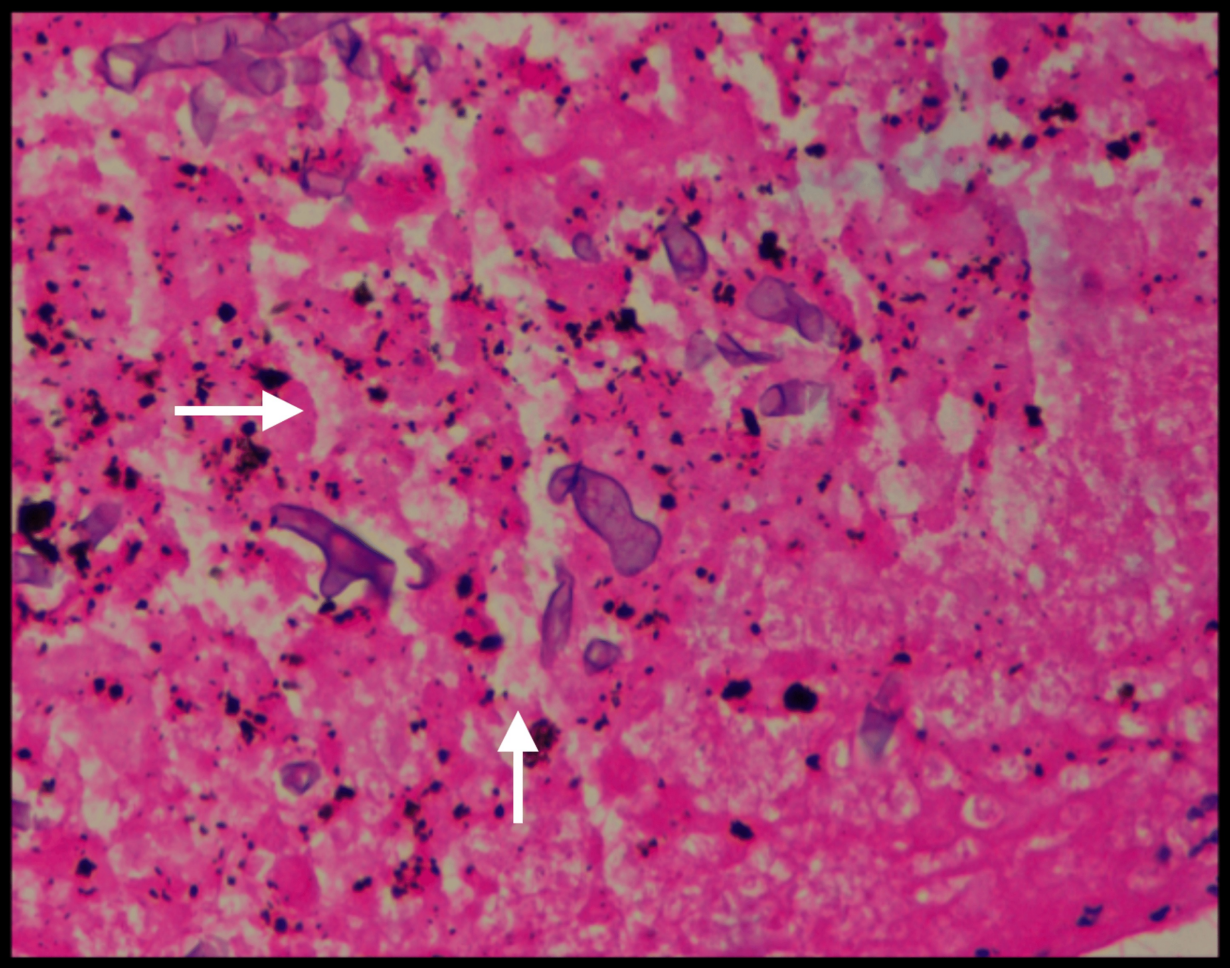
Histopathology showing fungal hyphae with acute angle branching, consistent with Mucorales (white arrows show Mucorales).

Histopathology of the bronchus biopsy confirmed the presence of pauci-septate fungal hyphae with acute angle branching, consistent with Mucorales (Figure 6).

Tragically, within a few days of diagnosis, the patient's condition deteriorated rapidly, leading to sudden worsening of breathlessness and respiratory failure, ultimately resulting in cardio-respiratory arrest.

## Discussion

Pulmonary mucormycosis is an exceedingly rare fungal infection but carries high mortality, with most cases occurring in immunocompromised hosts, including diabetes mellitus, hematological malignancy, or immunosuppressive drugs [5]. Most causative agents belong to the genus Mucorales, particularly *Rhizopus*, *Mucor*, and *Lichtheimia* species [6]. Its nonspecific presentation has features like fever, cough, hemoptysis, and chest pain, which clinically mimic other joint diseases, making early recognition hard [7]. In the most advanced stages, the cases of necrotizing pneumonia will worsen the clinical picture with dyspnea and cyanosis, especially in the late evolution [8]. High-resolution CT imaging of the thorax often shows nodules, cavitations, and pleural effusion, while definitive diagnosis is achieved by bronchoscopy with biopsy and culture [5]. Additionally, high-resolution CT imaging of the thorax might be the reverse halo sign, which is highly suggestive of this invasive fungal infection [9]. Molecular techniques, particularly polymerase chain reaction (PCR), are increasingly used to enhance diagnostic precision, thereby providing faster and more specific identification of the fungal species involved [9]. Management comprises surgical debridement and antifungal treatment with mostly amphotericin B, followed by consolidation on posaconazole or isavuconazole [10]. The angioinvasive nature of fungi may lead to extensive tissue necrosis and common resection of involved areas for sepsis control, which makes surgical debridement often life-saving [8]. The underlying predisposing factors need correction to enable the effective management of these patients, such as the improvement of glycemic control in a diabetic patient or reduction of immunosuppression when possible [5]. These have a poor outcome despite aggressive treatment, with mortality rates ranging from 30% to 70%, thus the importance of early detection and prompt intervention [11]. Factors that would lead to a more favorable outlook would be early diagnosis, initiation of appropriate antifungal therapy, and complete surgical resection of infected tissues [11]. This underlines increased clinical suspicion with earlier management ensuring improved outcomes, especially in high-risk groups like those post-hematopoietic stem cell transplantation or long-term corticosteroid therapy [12]. The prognosis in pulmonary mucormycosis is usually dismal but changes with early recognition and treatment. These are being studied now, and novel antifungal agents and combination therapy may, in the future, offer hope in giving better results [5]. Further, molecular diagnostic techniques and imaging modalities are being furthered to improve early detection and accurate identification of the causative agents, with potential reduction in diagnosis delays that significantly affect the survival of patients [9]. Therefore, more research and higher awareness among healthcare providers will assist in understanding and fighting this killer infection effectively [12].

## Conclusions

Pulmonary mucormycosis, though rare, is a life-threatening infection that demands increased clinical suspicion, particularly in immunocompromised hosts. Hence, there is a need for fast diagnosis and aggressive antifungal therapy or timely surgical interventions in the treatment of this potentially fatal infection. The development of new agents and therapeutic strategies is the direction for future research to offer better results to those under the influence of this terrible disease.
